# Galectins-1, -3 and -9 Are Present in Breast Milk and Have a Role in Early Life Development

**DOI:** 10.3390/nu14204338

**Published:** 2022-10-17

**Authors:** Karla Rio-Aige, Marina Girbal, Marta Selma-Royo, Anna Parra-Llorca, Sonia González, Cecilia Martínez-Costa, Margarida Castell, María Carmen Collado, Francisco J. Pérez-Cano, María J. Rodríguez-Lagunas

**Affiliations:** 1 Physiology Section, Department of Biochemistry and Physiology, Faculty of Pharmacy and Food Science, University of Barcelona (UB), 08028 Barcelona, Spain; 2 Nutrition and Food Safety Research Institute (INSA-UB), 08921 Santa Coloma de Gramenet, Spain; 3 Institute of Agrochemistry and Food Technology (IATA-CSIC), National Research Council, 46980 Valencia, Spain; 4 Neonatal Research Group, Health Research Institute La Fe, 46026 Valencia, Spain; 5 Department of Functional Biology, Faculty of Medicine, University of Oviedo, 33071 Oviedo, Spain; 6 Diet, Microbiota and Health Group, Instituto de Investigación Sanitaria del Principado de Asturias (DIMISA, ISPA), 33011 Oviedo, Spain; 7 Department of Pediatrics, INCLIVA Biomedical Research Institute, University of Valencia, Avenida Blasco Ibáñez 15-17, 46010 Valencia, Spain

**Keywords:** galectin, breast milk, umbilical cord plasma, maternal plasma

## Abstract

Galectins (Gal) are a family of conserved soluble proteins with high affinity for β-galactoside structures. They have been recognized as important proteins for successful pregnancy. However, little is known about their presence in breast milk and their role in early infancy. Gal-1, -3 and -9 concentrations were evaluated by Multiplex immunoassays in mother–infant pairs from the MAMI cohort in maternal plasma (MP) (*n* = 15) and umbilical cord plasma (UCP) (*n* = 15) at birth and in breast milk samples (*n* = 23) at days 7 and 15 postpartum. Data regarding mother and infant characteristics were collected. Gal-9 was present in a lower concentration range than Gal-1 and Gal-3 in plasma, specifically in UCP. A major finding in the current study is that Gal-1, -3 and -9 were detected for the first time in all the transitional breast milk samples and no differences were found when comparing the two breastfeeding time points. Finally, Gal levels were associated with some maternal and infant characteristics, such as gestational age, pregnancy weight gain, maternal diet, the gender, infant growth and infant infections. In conclusion, Gal levels seem to be involved in certain developmental aspects of early life.

## 1. Introduction

Galectins (Gals) were originally described as a group of proteins, produced by all cell types at varying levels, with high affinity to β-galactosides and high similarity in the sequence of the carbohydrate-binding sites, located in the carbohydrate-recognition domain (CRD) [1]. Later on, it was shown that Gals also interact with molecules that do not contain galactose and can also present non-carbohydrate-binding sites within the CRD [2]. According to the number and disposition of CRDs, Gals can be classified into three main groups: prototype, chimera and tandem. The prototype or mono Gals contain only one CRD, which are synthesized as monomers but often form homodimers. Gal-1, -2, -5, -7, -10, -11, -13, -14, and -15 belong to this group [3]. The only chimera-type Gal, Gal-3, is characterized by displaying a C-terminal CRD and a longer N-terminal domain. It is capable of dimerizing through the C terminal [4] or multimerizing through interactions between various Gal-3 N-terminal domains [5]. The tandem Gal, including Gal-4, -6, -8, -9 and -12, are comprised by two CRDs linked by a flexible peptide chain [3]. These two CRDs are homologous but recognize different glycan ligands [6].

Gals can have both immunosuppressive and immune regulatory functions, mainly depending on the cell type and the cell receptor they are binding to. Gal-1, -3 and -9 are some of the most abundant Gals, since they are expressed by a wide range of cells, including macrophages and epithelial cells [7]. Moreover, these Gals have been specifically related to pregnancy and birth. In newborns with inflammatory conditions, blood Gal-1, -9 (in amniotic infection syndrome) and Gal-3 (in neonatal sepsis) levels were increased compared to healthy infants [8]. For this reason, it is necessary to increase the knowledge regarding the implications of these components in both physiological- but also pathophysiological-related events.

The gene encoding for Gal-1 (*LGALS1*) was one of the most highly expressed in the decidua at term gestation [9]. Both *LGALS1* and *LGALS3* (Gal-3-encoding gene) had a higher expression in Cesarean section at term as compared to cases with spontaneous term labor, indicating an anti-inflammatory effect of Gals during pregnancy [9].

Gal-1, the most studied Gal in pregnancy, has been reported to have a critical role in the establishment of tolerance between the fetus and the mother, mediating apoptosis of activated CD8 + T cells, T helper (Th) 1 and Th17 cells. For this reason, a decreased expression of Gal-1 was positively correlated with failed pregnancies [10,11,12]. Dysregulation of Gal-1 has also been associated with pre-eclampsia, and with increased or decreased placental expression in patients with late and early onset pre-eclampsia, respectively [13]. Upregulation of Gal-1 has also been linked to breast cancer [14].

Likewise, Gal-3 downregulation in the placenta, leading to placental dysfunction, was correlated with fetal growth restriction (FGR) [15]. In addition, Gal-3 knock-out mice in the endometrium had substantially lower embryo implantation [16]. Moreover, in patients with gestational diabetes mellitus, Gal-3 expression was increased in maternal blood and placental tissue and decreased in cord blood when compared to healthy patients [17].

Gal-9 has been mainly related to the regulation of natural killer (NK) cells function to promote maternal–fetal tolerance in early pregnancy. Gal-9 levels are elevated during pregnancy as compared to healthy non-pregnant controls and decrease at parturition, having an immunoregulatory effect [18].

Since breast milk (BM) contains abundant human milk oligosaccharides (HMO), which are a combination of the five monosaccharides glucose, galactose, fructose, N-acetyl-glucosamine and sialic acid [19], it seems plausible that Gals may also be present in BM. Only two previous studies have focused on this hypothesis. Noll et al. (2016) reported that Gals were not detected in human BM when using a western blot analysis. Nevertheless, the authors demonstrated that human milk glycans (HMG) were selectively bound by Gals, suggesting that these interactions could play a role in infant immunity [20]. In another study, Coscia et al. (2012) detected low levels of Gal-7 in colostrum from healthy mothers through protein identification by tandem mass spectrometry [21].

Despite the increasing literature on the role of Gals on pregnancy, to our knowledge nothing is known about Gal-1, -3 and -9 presence in BM. For that reason, we evaluated the presence and concentrations of these Gals in human plasma and BM samples from two independent subgroups of mother–infant pairs within the MAMI cohort [22]. The current study had three main goals consisting of: characterizing the Gal-1,-3 and -9 levels in maternal plasma and arterial umbilical cord plasma on the delivery day; detecting whether Gal-1,-3 and -9 are present in human BM at two points of the transitional stage BM; and identifying potential associated perinatal factors influencing galectin composition of BM.

## 2. Materials and Methods

### 2.1. Cohort and Study Subjects

A total of 15 healthy mother–infant pairs for plasma analysis, and an extended group of 23 healthy mother–infant pairs for BM analysis, were included into this sub-study from the MAMI cohort (Clinical trial Registry NCT03552939) [22]. Maternal Weight and height, and also the infant’s growth parameters, were longitudinally recorded in medical consultation. For infants, BMI *z* score was calculated with the WHO Anthro software (www.who.int/childgrowth/software/en/); accessed on 1 June 2020.

The 140-item Food Frequency Questionnaire (FFQ) was used by a nutritionist to collect the maternal dietary records during the first week after birth. FFQ information was analyzed for the energy and daily intake of macro- and micronutrients, as commented in previous articles [23,24]. Regarding the variable “type of diet” of the present study, the previous results of the MAMI cohort allowed us to separate the mothers into two dietary clusters. One cluster group was characterized by higher intake of fiber, vegetal protein and polyphenols, and the second cluster was characterized by higher saturated fatty acid and animal protein levels [23,25].

### 2.2. Maternal Plasma and Arterial Umbilical Cord Sampling

Maternal blood was obtained from 15 mothers and collected in sterile containers into anticoagulant (EDTA)-treated tubes immediately prior to delivery (20–30 min before expulsion or before incision in C-section deliveries), as in previous approaches [24]. Arterial umbilical cord blood was obtained from 15 infants and also collected in EDTA tubes immediately, in the delivery room, after delivery of the placenta, coinciding with the control blood gases performed on the cord (5 min after birth and immediately after cord clamping). Blood processing was carried out in FISABIO-GVA Biobank. In brief, blood cells were removed from plasma by centrifugation at 1500× *g* for 10 min at 4 °C. The plasma was centrifuged again at 2500× *g* for 10 min at room temperature to deplete platelets in the plasma. The resulting supernatant was aliquoted and stored at −80 °C until further analysis, corresponding on the one hand to the maternal plasma (MP) and on the other hand to the umbilical cord plasma (UCP) [24].

### 2.3. Breast Milk Samples

BM samples for this study were obtained at 7 and 15 days after birth in the morning before lactation [23,25]. In brief, breast skin was cleaned with a solution of 0.5% chlorhexidine to collect samples with a sterile pumper, discarding the first drops to normalize the collection. Aqueous phase was obtained by centrifugation at 2000× *g* for 15 min at 4 °C. Finally, samples were frozen at −80 °C for latter analysis.

### 2.4. Determination of Gal Concentrations

The quantification of Gals (Gal-1, Gal-3 and Gal-9) was performed by ProcartaPlex^TM^ Multiplex immunoassay (Thermo Fisher Scientific, Vienna, Austria) using a Gal-1 Human ProcartaPlex^TM^ Simplex Kit, a Gal-3 Human ProcartaPlex^TM^ Simplex Kit and a Gal-9 Human ProcartaPlex^TM^ Simplex Kit. To develop the analysis, the manufacturer instructions were followed as in previous studies [26]. The plate was run on a Luminex Instrument and analyzed in a ProcartaPlex Analyst Software (MAGPIX^®^ analyzer, Luminex Corporation) at the Flow Cytometry Unit of the Scientific and Technological Centres of the University of Barcelona (CCiT-UB). The test interval was as follows: 394.46–615,700.00 pg/mL for Gal-1; 439.45–1,800,000.00 pg/mL for Gal-3 and 2.22–9100 pg/mL for Gal-9. One outlier UCP sample was discarded from the Gal study.

### 2.5. Data Processing and Statistical Analysis

Statistical analysis was performed using IBM SPSS Statistics for Windows, version 22 (IBM Corp., Armonk, NY, USA) and RStudio version 4.2.0 (R foundation, Vienna, Austria) (http://www.rstudio.com/ (accessed on 20 April 2022)). Results are expressed as mean ± SEM, unless otherwise specified. Shapiro–Wilk and Levene’s tests were used to determine normality and homogeneity of data variance, respectively. When data were normally distributed, Student’s *t* test was used to assess significant differences between groups. When variables were not normally distributed, non-parametric tests were used, as Kruskal–Wallis and Mann–Whitney U tests. The spearman correlation coefficient was used to search for correlation between variables. Clustering of the individuals was analyzed by non-metric multi-dimensional scaling (NMDS) in Rstudio using the R package vegan (Community Ecology Package. R package version 2.4-6 (https://CRAN.R-project.org/package=vegan) (accessed on 11 October 2022) [27]. In the NMDS plot the complex dimensional data was represented in 2 dimensions to highlight the similarities between samples in terms of their Gal composition. In these term, the closer two samples are to each other, the more similar they are. Furthermore, more information was overlaid on ordination NMDS plot with the function “envfit” to represent vectors onto the plot. Longer vectors mean a stronger association with the samples in that direction. *p* < 0.05 was considered significant for all analyses performed.

## 3. Results

### 3.1. Galectins in Plasma from Mother–Infant Pairs

#### 3.1.1. Clinical Characteristics

The assessment of Gal-1, -3 and -9 concentrations in maternal plasma (MP) and umbilical cord plasma (UCP) was performed in a subgroup of the MAMI birth cohort [22] consisting of 15 mother–infant pairs. Maternal and infant characteristics for this subgroup are presented in Table 1.

#### 3.1.2. Galectins Concentration in MP and UCP

**Figure 1:**
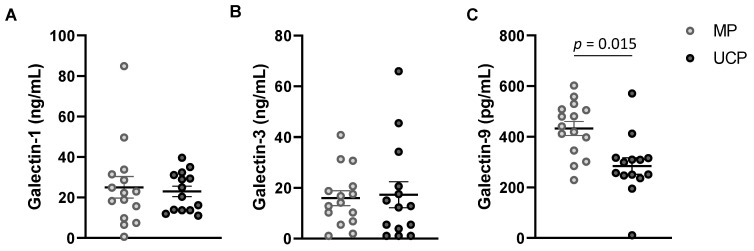
Gal-1 (**A**), Gal-3 (**B**) and Gal-9 (**C**) concentrations (pg/mL) in maternal plasma (MP) and umbilical cord plasma (UCP). *p* values were obtained using Mann–Whitney U test.

All three Gals (1,3,9) were detected in both MP and UCP samples. Gal-1 and -3 were present in plasma at concentrations up to 60–80 ng/mL, while Gal-9 was present in a lower concentration range, up to 600 pg/mL. For Gal-1 and -3, no significant difference was found between the concentrations in MP and UCP (Figure 1A,B). Conversely, Gal-9 levels were significantly higher in MP than in UCP (Figure 1C).

A Spearman correlation of pairwise comparison showed that Gal-1,-3,-9 concentrations were not correlated in mother–infant pairs, suggesting that the maternal–fetal transfer of Gals might not be the only factor involved in determining the UCP values (Figure S1).

#### 3.1.3. Association of Maternal and Infant Factors with Galectins Concentrations

Maternal and infant factors (Table 1) were assessed to elucidate potential correlations with Gal concentrations in MP and UCP. Although a low number of samples were analyzed, multiple variables were identified with a significant effect on Gal concentrations, i.e., infant gender, gestational age and infant intake of antibiotics from parturition to 6 months.

**Figure 2:**
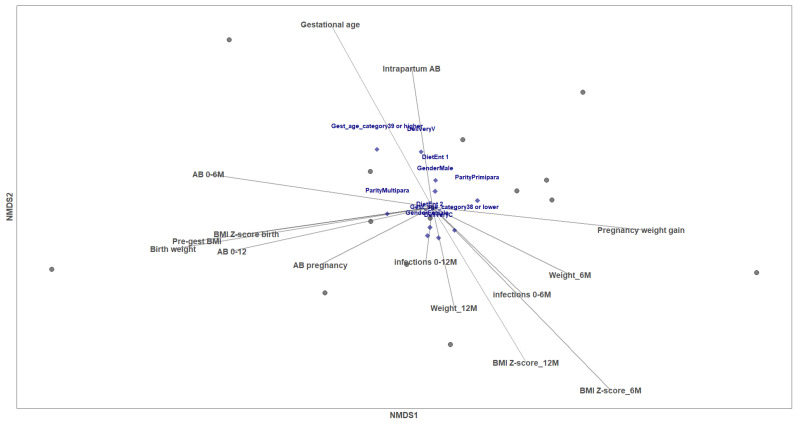
Maternal and infant characteristics influencing Gal concentrations in MP. Non-metric multi-dimensional scaling (NMDS) for the Gal concentrations based on the Bray–Curtis distance (stress: 0.023). Each point represents a mother taking into account the joint Gal-1,-3 and -9 levels. Categorical variables are written in blue and continuous variables are represented by vectors.

Among the influencing infant and mother-related variables analyzed by non-metric multi-dimensional scaling (NMDS) analysis (gestational age, mode of delivery, parity, type of diet, gender, baby’s weight and BMI *z* score, antibiotic intake during pregnancy and/or exposure at the delivery day and neonate’s antibiotic intake and infections up to 12 months), gestational age and BMI *z* score at 6th month were significantly correlated with the NMDS ordination of the mothers based on Gal levels in MP (envfit R^2^ = 0.5267, *p* value = 0.019; envfit R^2^ = 0.6190, *p* value = 0.006, respectively) (Figure 2). NMDS analysis for the same variables and the Gal concentrations in UCP at time of birth showed that none of the assessed vectors and factors had a significant effect on the ordination of UCP Gal levels (Figure S2).

As for the effect of gender, a significantly higher concentration of Gal-9 in MP of mothers having male babies was observed (Table 2).

**Figure 3:**
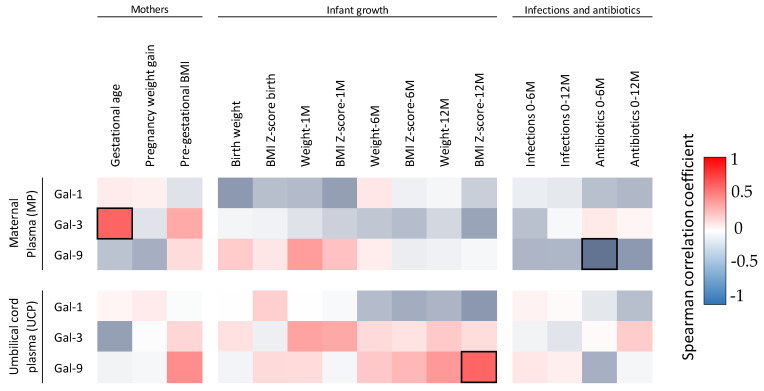
Correlations between Gal levels and maternal and infant characteristics. The Spearman correlation coefficient is represented in the heat map following the color in the legend. Bold frames represent correlations with statistical significance (*p* < 0.05). M, month.

A positive significant Spearman correlation was also found between gestational age and Gal-3 in MP and between the infants’ BMI *z* score at 12 months and Gal-9 in UCP. A negative significant Spearman correlation was also found between infant antibiotic treatment during the first 6 months of life and Gal-9 concentrations in MP (Figure 3).

### 3.2. Galectins Presence in Breast Milk

#### 3.2.1. Clinical Characteristics

Furthermore, we aimed to characterize the Gal levels of breast milk at 2 sampling time points within the transitional stage, at day 7 (d7) and at day 15 (d15) from 23 mothers belonging to the MAMI cohort [22]. The demographic data of the mothers and their respective infants is described in Table 3.

#### 3.2.2. Galectin Levels in Transitional Milk at Day 7 and 15

Interestingly, Gal-1, Gal-3 and Gal-9 were all detected at both days, with Gal-9 being the Gal present at the lowest concentration (Table 4). No significant difference was found between Gal levels at day 7 and day 15 in BM (Table 4), however, the levels of all three galectins at d7 were highly positively correlated with the levels at d15 (Figure S3).

#### 3.2.3. Perinatal Factors Shaping Galectin Levels in Breastmilk

**Figure 4:**
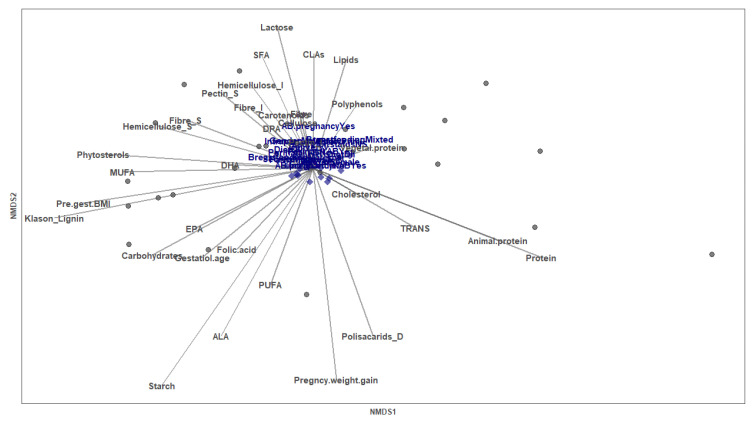
Perinatal factors shaping Gal levels in breastmilk at d7. Non-metric multi-dimensional scaling (NMDS) for the Gal concentrations based on the Bray–Curtis distance (stress: 0.027). Each point represents a mother (*n* = 23). Categorical variables are written in blue and continuous variables are represented by vectors.

Among the influencing perinatal qualitative variables analyzed by NMDS analysis (type of delivery, parity, breastfeeding (maternal or mixed), type of diet, gender of the baby and antibiotic intake during pregnancy and/or at the delivery day), none of them were significantly correlated with the NMDS ordination of the mothers based on Gal levels at day 7. However, the vector of the dietary component starch was highly related with the position of the individuals (envfit R^2^ = 0.3063, *p* value = 0.019) and the pregnancy weight gain tended to be correlated (envfit R^2^ = 0.2455, *p* value = 0.065) (Figure 4). Despite the global influence of the starch in the NMDS, no Spearman correlation was found between this dietary component and the Gals separately (Figure S4). As for the effect of the pre-gestational BMI, a negative significant Spearman correlation was found with the Gal-1 from d7 (Figure S5).

**Figure 5:**
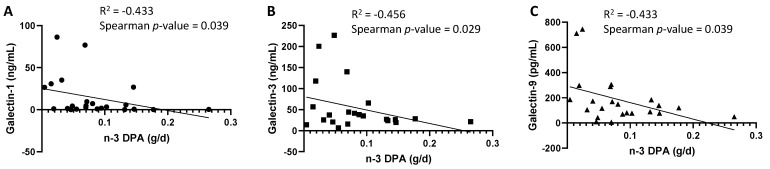
Correlations between the intake of n-3 Docosapentaenoic acid (DPA) and Gal-1 (**A**), Gal-3 (**B**) and Gal-9 (**C**) concentrations (pg/mL) from breast milk at day 15.

Regarding the influence of perinatal variables on BM Gals at day 15, including dietary intake, no influence was found in the NMDS analysis (Figure S6). However, significant negative Spearman correlations were found between all galectins from day 15 with the intake of the n-3 Docosapentaenoic acid (DPA) (Figure 5).

#### 3.2.4. Infant Factors Associated with Breast Milk Galectin Levels

**Figure 6:**
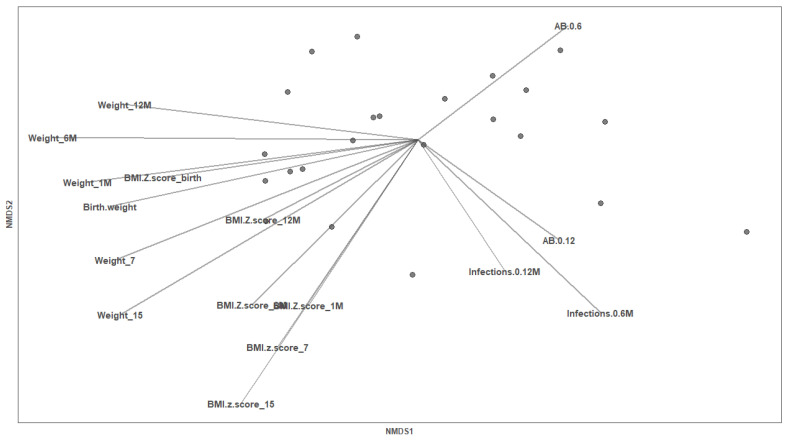
Infant factors associated with breast milk Gals at day 7. Non-metric multi-dimensional scaling (NMDS) for the Gal concentrations based on the Bray–Curtis distance (stress: 0.027). Each point represents a mother (*n* = 23). Continuous variables are represented by vectors. M, month; AB, antibiotic.

**Figure 7:**
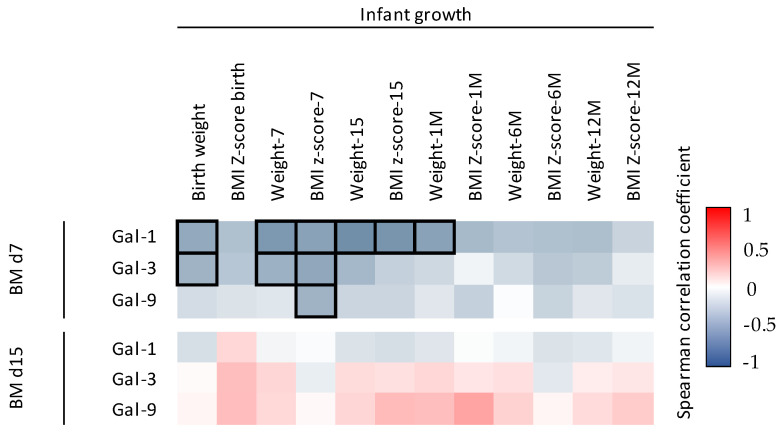
Correlations between Gal levels and infant growth parameters. The Spearman correlation coefficient is represented in the heat map following the color in the legend. Bold frames represent correlations with statistical significance (*p* < 0.05). BM, breast milk; M, month.

**Figure 8:**
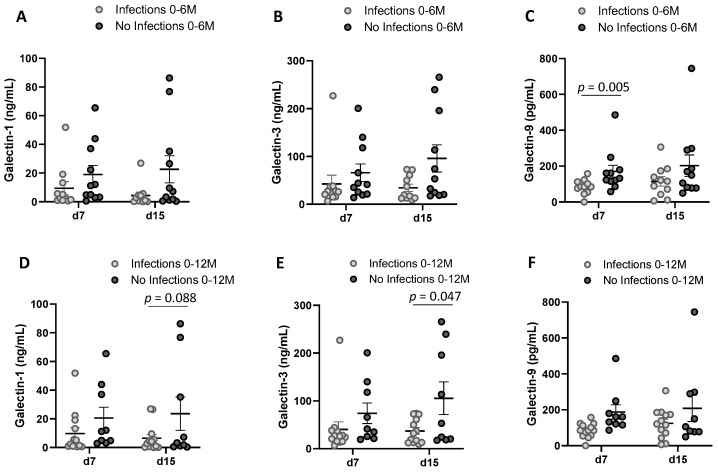
Breast milk Gal levels from mothers whose child did not have an infection or had some infection from birth to 6 months (*n* = 11, *n* = 11, respectively) (**A**–**C**) and from birth to 12 months (*n* = 9, *n* = 13, respectively) (**D**–**F**). Results are expressed as mean ± SEM. *p* values were calculated with Mann–Whitney U test. Infection includes both gastrointestinal and respiratory conditions.

To investigate the association between Gal levels and infant factors (weight, BMI *z* score, antibiotic intake and infections), we also performed the NMDS analysis with the Gal levels at day 7 and day 15, separately. Multiple variables were identified as potential associated factors with the BM Gals at day 7 (Figure 6), whereas no variable was related with the BM Gals at day 15 (Figure S7). The weight at birth, at 7 days, at 15 days, at 1 month, at 6 months, at 12 months, the BMI *z* score at day 7 and 15 and the infections from parturition to 6 months influenced the ordination of the individuals (envfit R^2^ = 0.3113, *p* value = 0.037; envfit R^2^ = 0.3495, *p* value = 0.022; envfit R^2^ = 0.4172, *p* value = 0.007; envfit R^2^ = 0.3428, *p* value = 0.025; envfit R^2^ = 0.4085, *p* value = 0.016; envfit R^2^ = 0.2696, *p* value = 0.065; envfit R^2^ = 0.2668, *p* value = 0.077; envfit R^2^ = 0.4319, *p* value = 0.006; envfit R^2^ = 0.2466, *p* value = 0.085, respectively) (Figure 6). In line with these results, Gal-1, Gal-3 and Gal-9 concentrations at day 7 were significantly negatively correlated with some infant growth parameters (Figure 7). Regarding the infant infections, the breast milk at both sampling days showed higher levels of Gals in the group of mothers with infants without infections compared to the group with infections (Figure 8), being significantly different from Gal-9 at day 7 (Figure 8C) and Gal-3 at day 15 (Figure 8E).

## 4. Discussion

The role of galectins (Gals) in pregnancy and birth has gained interest in the past few decades, specifically, Gal-1, -3 and -9; however little is known about its relationship with some maternal, prenatal and postnatal factors. In the present study, we performed a characterization of these Gals in plasma at the delivery day, and in breast milk samples at two dates during the transitional breastfeeding period. To our knowledge, this is the first time that these Gals are found in breast milk and are associated with infant health parameters.

In order to support a successful pregnancy, immune factors participate in coordinated communications between the mother and the fetus [28,29]. In this line, in the present study, we aimed to establish the concentration of Gal-1, -3 and -9 in plasma from maternal-neonatal pairs at birth and to associate them with the perinatal factors, by using 15 samples from a mother–infant birth cohort in the Spanish Mediterranean area (MAMI) [22]. All three Gals were detected in MP and UCP. To our knowledge, previous publications have not focused on comparative levels of these Gals in MP and UCP in healthy term newborns. Gal-1 and -3 concentrations were 100-fold higher in both plasmas than Gal-9, in line with previous publications [12,30,31]. Natural passive immunization is conferred by the mothers to the infants through the transplacental pathway during the gestation period: several articles have confirmed the transmission of IgG by the FcRn receptor [32,33]. Moreover, we previously observed with a larger group within the same cohort that the IgG subtypes (IgG1, G2, G3 and G4) were highly correlated between MP and UCP [24]. However, no significant Spearman correlation was found for Gal-1, -3 and -9 in MP and UCP pairs in the present study, suggesting that the maternal–infant Gal transmission through the placenta might not be the only mechanism involved. In fact, Gal-3 has also been described to be present in the amniotic fluid ([34]), the composition of which comes from both maternal and fetal origins. However, there is no specific evidence so far of the ability of the fetuses or the neonates to produce galectins. Overall, this fact could explain the lack of correlations between UCP and MP galectin levels in the present work and deserves to be further studied.

Plasma Gal levels have been reported to highly fluctuate in relationship to inflammatory conditions. He et al. (2016) reported similar levels for Gal-1, -3 and -9 after a large artery atherosclerotic stroke [35]. Enninga et al. (2017) obtained similar MP Gal-9 levels to the current study, which were maintained during pregnancy and until delivery, but dropped significantly postpartum [18]. Tirado-González et al. (2013) reported increasing levels of MP Gal-1 during pregnancy, up to a level around 80 ng/mL at the time of delivery, in contrast to < 25 ng/mL for non-pregnant controls [12]. Linden et al. (2013) showed that Gal-3 levels in UCP changed as a function of gestational age: neonates born earlier than 28 weeks or later than 37 had lower levels than those born between 28–36 weeks [36]. While in the current publication a significant correlation between gestational age and Gal-3 in UCP was not found, this factor was significantly positively correlated with Gal-3 levels in MP at the time of birth.

The finding that Gal-9 concentration in MP at birth was significantly higher for women giving birth to male fetuses than those giving birth to females corroborates previous findings [18]. Previous studies have reported the role of Gal-9 in establishing tolerance between the mother and the fetus, as well as its anti-inflammatory effect [18,37,38]. In this study, Gal-9 concentration in MP at birth was negatively correlated with infections and intake of antibiotics during the first year postpartum, with intake of antibiotics during the first 6-months after birth being the only significant correlation to Gal-9 levels. This data suggests that infants from mothers with lower plasma concentrations of Gal-9 may be more susceptible to infections and need antibiotic intake during the first year of life.

To date, the presence of Gals in breast milk remains elusive, despite the physiological importance they could have in the infant health. On top of that, previous studies have reported an absence of Gals in human milk (from an unknown breastfeeding phase) when using western blot analyses [20] and when performing a proteome mapping of human milk proteins [39]. Nevertheless, Gal-7 has been detected in a low concentration in colostrum samples from healthy mothers through protein identification by tandem mass spectrometry [21]. Thus, the strong point of the present study is that Gal-1, -3 and -9 were detected in the transitional breast milk from all the mothers at 14.55 ng/mL, 60.15 ng/mL and 0.17 ng/mL concentrations, respectively, performing the immunoassay with magnetic beads in the Luminex instrument platform. Physiologically, some BM immune components decrease along the breastfeeding stages (colostrum, transitional milk and mature milk), specifically the IgA [40,41,42], which is the Ig found in the highest proportion in BM [43]. Nevertheless, we found similar levels comparing the two breastfeeding points (d7 and d15), indicating that Gals in BM could be more stable in time than other immune molecules.

Regardless of the similar Gal levels found at both time points, Gals at the beginning of the transitional stage, i.e., at day 7, showed stronger Spearman correlations to perinatal factors than day 15 samples. With regard to the maternal influencing factors, the overall dietary composition was not shaping the Gals profile however the highest influence due to diet was originated from starch and DPA intake. Early in life, a change in starch concentration in maternal diet has been related to a modulation of the lipid metabolism of newborn piglets [44] and a modulation of the milk production in cows [45]. DPA is an essential omega-3 polyunsaturated fatty acid related with several beneficial anti-inflammatory properties [46] and with potential health benefits for the infant development [47]. Further studies are needed to confirm how maternal diet could affect BM Gal levels, specifically in the beginning of breastfeeding.

With respect to the infant variables, the infant’s growth-related parameters were highly associated with the BM Gals concentrations, specifically to Gal-1 and 3 levels at day 7. The higher the Gal-1 and 3 at day 7, the lower the weight and BMI *z* score during all the time period comprised from birth to 1 month. Previous studies have shown that multiple other milk components, such as fats, carbohydrates and proteins [48,49], leptin, adiponectin, ghrelin [50] and short chain fatty acids [51] are related to the growth of infants. New studies are required to understand the physiological relevance of BM Gals in the infant gut and, in turn, in the infant growth.

The joint analysis of all the factors influencing Gal-1, -3 and -9 concentrations in plasma and milk indicates that levels could be related with weight and immunity parameters. Interestingly, Gal-9 levels in MP at birth and in BM at day 7 postpartum are negatively correlated with infections and antibiotic intake during the first 12 months. While in BM a significant negative correlation was found for Gal-9 and infections during the first 12 months, in MP, the correlation was significant and negative for antibiotic intake during the first 6 months. These results suggest a possible role of Gal-9 in early life protection against infection.

Comparison of Gal concentrations showed that Gal-1 concentration was about 1.5-fold lower in MP than in BM, Gal-3 was about 4-fold higher and Gal-9 was about 2.5-fold lower. A tolerogenic and anti-inflammatory effect has been associated with Gal-9 in pregnancy. The fact that it dropped significantly at 6 weeks postpartum could be in line with the fact that the concentration is lower in BM than in MP in our results, contributing to a higher Th1/Th2 balance needed to a correct immunological response in the infancy [52]. All these data reinforce the hypothesis that Gal levels, and specifically Gal-9 levels after birth, have a role in the infant’s immunity during the first year of life, with higher levels of Gal-9 helping prevent infant’s infections and an associated antibiotic intake during this period of time.

The limitations of this study include sample size and the absence of plasma-breast milk matching samples. These facts could have affected the statistical power and the strength of the associations with the perinatal outcomes, respectively, and deserve to be considered in the future.

## 5. Conclusions

The results of the study demonstrated that Gal-1, -3 and -9 are present both in plasma at birth and in BM at transitional stage. Furthermore, Gal levels were not correlated in MP–UCP pairs, and Gal-9 was significantly lower in UCP with respect to the MP. Besides, Gal concentrations were similar when comparing both breastfeeding days corresponding to the beginning and the end of the transitional stage.

**Figure 9:**
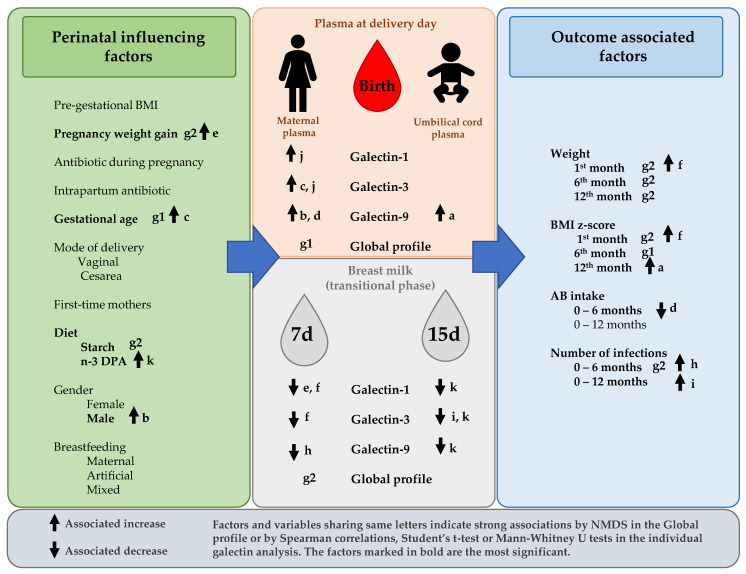
Summary of the results. Factors and variables sharing same letters indicate strong associations by NMDS in the Global profile or by Spearman correlations, Student’s *t* test of Mann–Whitney U tests in the individual galectin analysis. The factors marked in bold are the most significant. DPA, Docosapentaenoic acid.

Moreover, results showed that Gal levels were associated with some maternal and infant characteristics. Specifically, the gestational age and the gender were related to the MP Gal levels and the dietary starch and DPA on the BM Gal levels. Moreover, some other factors were also related, highlighting the infant antibiotic use with the MP Gal levels and the infant growth and infections with the BM Gals, specifically at the beginning of the transitional phase (Figure 9).

Finally, the understanding of the presence of Gals in BM and the mother’s plasma during labor and the underlying molecular mechanisms could contribute to the design of interventions in order to sustain infant health and to prevent infant infections.

## Figures and Tables

**Table 1:** Mother–infant birth cohort characteristics.

Maternal Characteristics	*n* = 15
Pre-gestational BMI (kg/m^2^), mean ± SEM	24.36 ± 1.21
Pregnancy weight gain (kg), mean ± SEM	11.29 ± 1.09
Antibiotic during pregnancy, yes (%)	4 (26.67)
Intrapartum antibiotic, yes (%)	10 (66.67)
Gestational age (weeks), mean ± SEM	38.2 ± 0.30
Mode of delivery: vaginal birth, yes (%)	5 (33.33)
First time mothers, yes (%)	8 (53.3)
Gestational diabetes mellitus (GDM), yes (%)	2 (13.33)
Infant Characteristics	*n* = 15
Gender: Female, yes (%)	5 (33.33)
Body weight gain from birth to the 1st month (mg/day)	32.11 ± 2.57
Birth WFL *z* score, mean ± SEM	−0.29 ± 0.28
Weight (kg), mean ± SEM	
Birth	3.06 ± 0.11
1st month	4.03 ± 0.15
6th month	7.68 ± 0.15
12th month	9.56 ± 0.23
BMI *z* score, mean ± SEM	
Birth	−0.5 ± 0.27
1st month	−0.70 ± 0.34
6th month	−0.35 ± 0.19
12th month	0.13 ± 0.23

Maternal (*n* = 15) and infant (*n* = 15) characteristics associated with maternal and umbilical cord plasma samples (MP and UCP, respectively). BMI, body mass index. SEM, standard error of the mean.

**Table 2:** Galectins concentration based on neonate’s gender.

	Female	Male	*p*
MP	*n* = 5	*n* = 10	
Gal-1 (ng/mL)	16.10 ± 5.28	29.53 ± 7.32	0.327
Gal-3 (ng/mL)	11.47 ± 3.43	18.22 ± 3.95	0.426
Gal-9 (pg/mL)	350 ± 26	475 ± 32	0.020 *
UCP	*n* = 5	*n* = 9	
Gal-1 (ng/mL)	20.69 ± 4.16	24.32 ± 3.41	0.317
Gal-3 (ng/mL)	16.94 ± 7.75	17.51 ± 7.03	0.789
Gal-9 (pg/mL)	263 ± 89	296 ± 20	0.205

Gal-1, -3 and -9 concentrations in MP and UCP. Data shown is expressed as mean ± S.E.M. Samples were classified based on the gender of the baby. *p* values were calculated using the Mann–Whitney U test. * *p* value < 0.05.

**Table 3:** Mother–infant birth cohort characteristics.

Maternal Characteristics	*n* = 23
Pre-gestational BMI (Kg/m^2^), mean ± SEM	22.72 ± 0.92
Pregnancy weight gain (Kg), mean ± SEM	11.94 ± 1.13
Antibiotic during pregnancy, yes (%)	8 (34.78)
Intrapartum antibiotic, yes (%)	10 (43.48)
Perinatal antibiotic, yes (%)	14 (60.87)
Gestational age (weeks), mean ± SEM	39.29 ± 0.27
Mode of delivery: vaginal birth, yes (%)	15 (65.22)
First time mothers, yes (%)	10 (43.48)
Gestational diabetes mellitus (GDM), yes (%)	2 (8.69)
Infant Characteristics	*n* = 23
Gender: Female, yes (%)	12 (52.17)
Body weight gain from birth to the 1st month (mg/day)	25.33 ± 1.97
Birth WFL *z* score, mean ± SEM	−0.20 ± 0.19
Weight (kg), mean ± SEM	
Birth	3.18 ± 0.11
7th day	3.17 ± 0.09
15th day	3.40 ± 0.08
1st month	3.98 ± 0.11
6th month	7.41 ± 0.19
12th month	9.47 ± 0.25
BMI *z* score at birth, mean ± SEM	
Birth	−0.29 ± 0.20
7th day	−0.55 ± 0.22
15th day	−0.55 ± 0.19
1st month	−0.71 ± 0.21
6th month	−0.08 ± 0.16
12th month	0.39 ± 0.20

Maternal (*n* = 23) and infant (*n* = 23) characteristics associated to breast milk samples.

**Table 4:** Gal levels in breast milk at day 7 and 15.

	d7, *n* = 23	d15, *n* = 23	*p*
Gal-1 (ng/mL)	14.88 ± 3.86	14.22 ± 4.98	0.253
Gal-3 (ng/mL)	54.25 ± 12.43	66.05 ± 15.19	0.869
Gal-9 (pg/mL)	149.41 ± 29.61	182.73 ± 39.89	0.645

Data shown is expressed as mean ± S.E.M. Mann–Whitney U test was used to determine significant differences between days 7 and 15 of breastfeeding.

## Data Availability

Not applicable.

## Supplementary Materials

The following supporting information can be downloaded at: https://www.mdpi.com/article/10.3390/nu14204338/s1, Figure S1. Correlations between galectin levels in maternal plasma (MP) and umbilical cord plasma (UCP) at birth, Figure S2. Maternal and infant characteristics influencing galectin concentrations in UCP. Figure S3. Correlations between BM galectin levels at day 7 and at day 15, Figure S4. Correlations between BM galectin levels at day 7 and at day 15 with the maternal dietary intakes, Figure S5. Correlations between BM galectin levels at day 7 and at day 15 with some maternal factors, Figure S6. Perinatal factors shaping galectin levels in breastmilk at d15, Figure S7. Perinatal factors shaping galectin levels in breastmilk at d15.
