# Supplementary material for: Galectins-1, -3 and -9 Are Present in Breast Milk and Have a Role in Early Life Development
Source: Nutrients. 2022 Oct 17;14(20):4338. doi: 10.3390/nu14204338 (PMC9611974; doi:10.3390/nu14204338)
Supplement: Supplementary file 1 [file nutrients-14-04338-s001.zip › nutrients-1969336-supplementary.pdf]

Supplementary Data

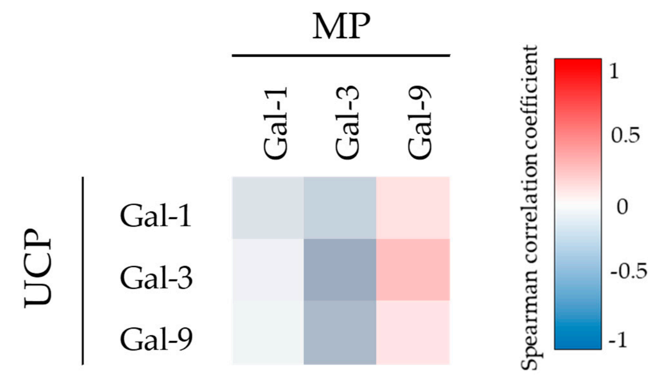

Supplementary Figure S1. Correlations between galectin levels in maternal plasma (MP) and umbilical cord plasma (UCP) at birth. The Spearman correlation coefficient is represented in the heat map following the color in the legend. Bold frames represent correlations with statistical significance ($p < 0.05$).

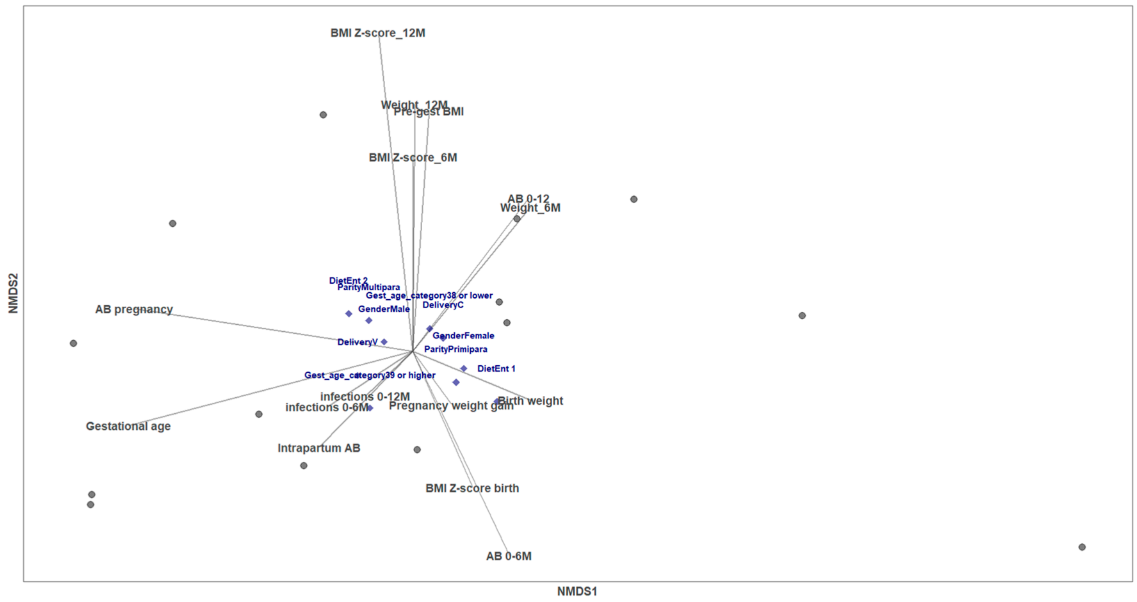

Supplementary Figure S2. Maternal and infant characteristics influencing galectin concentrations in UCP. Non-metric multi-dimensional scaling (NMDS) for the galectin concentrations based on the Bray-Curtis distance (stress: 0.022). Each point represents a mother taking into account the joint gal-1,-3 and -9 levels. Categorical variables are written in blue and continuous variables are represented by vectors.

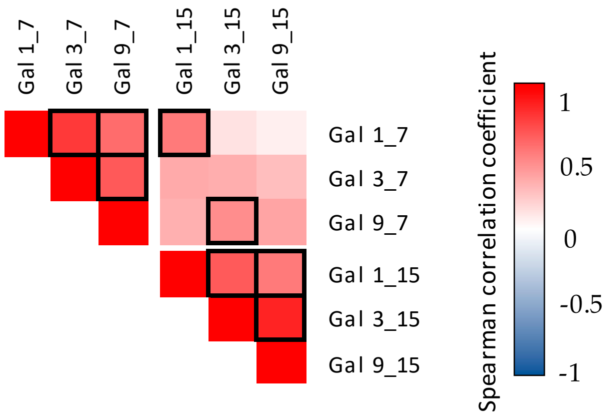

Supplementary Figure S3. Correlations between BM galectin levels at day 7 and at day 15. The Spearman correlation coefficient is represented in the heat map following the color in the legend. Bold frames represent correlations with statistical significance ($p < 0.05$).

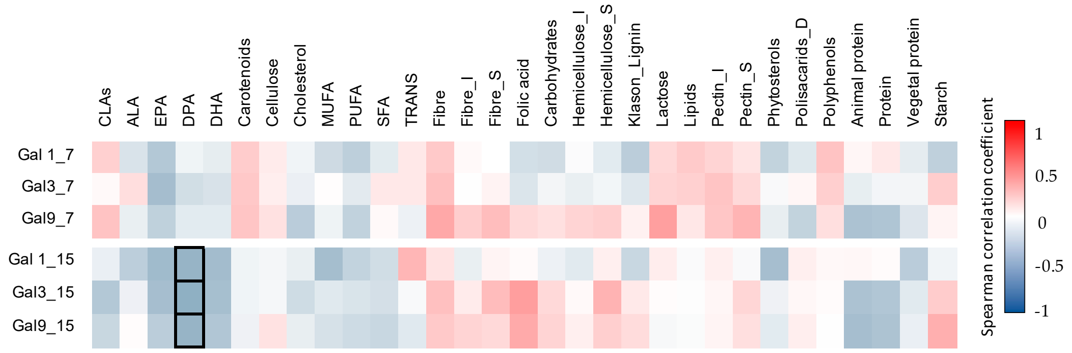

Supplementary Figure S4. Correlations between BM galectin levels at day 7 and at day 15 with the maternal dietary intakes. The Spearman correlation coefficient is represented in the heat map following the color in the legend. Bold frames represent correlations with statistical significance ($p < 0.05$).

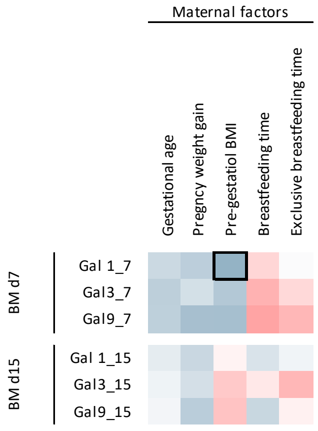

Supplementary Figure S5. Correlations between BM galectin levels at day 7 and at day 15 with some maternal factors. The Spearman correlation coefficient is represented in the heat map following the color in the legend. Bold frames represent correlations with statistical significance ($p < 0.05$).

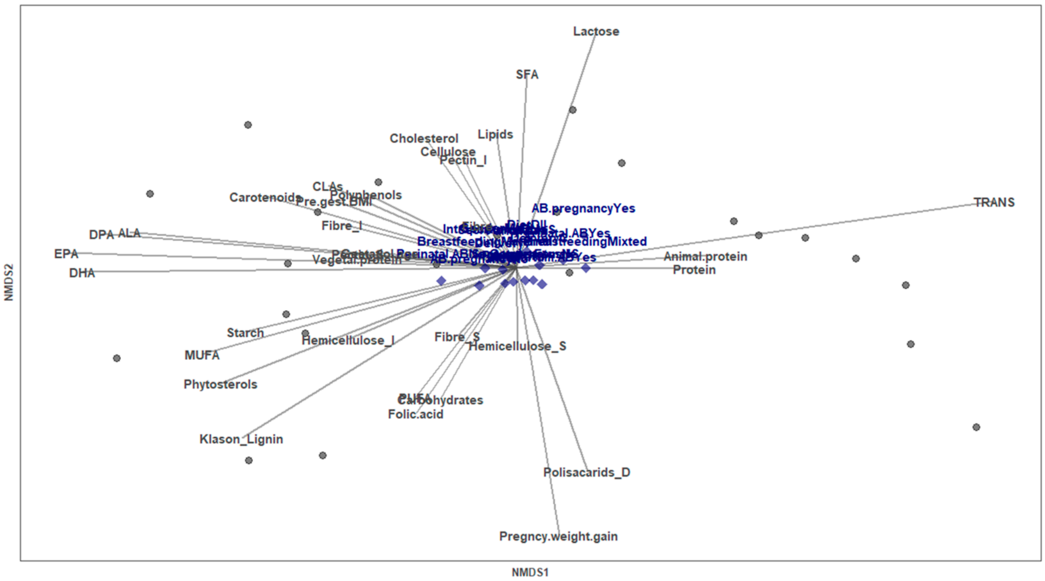

Supplementary Figure S6. Perinatal factors shaping galectin levels in breastmilk at d15. Non-metric multi-dimensional scaling (NMDS) for the galectin concentrations based on the Bray-Curtis distance (stress: 0.027). Each point represents a mother (n = 23). Categorical variables are written in blue and continuous variables are represented by vectors.

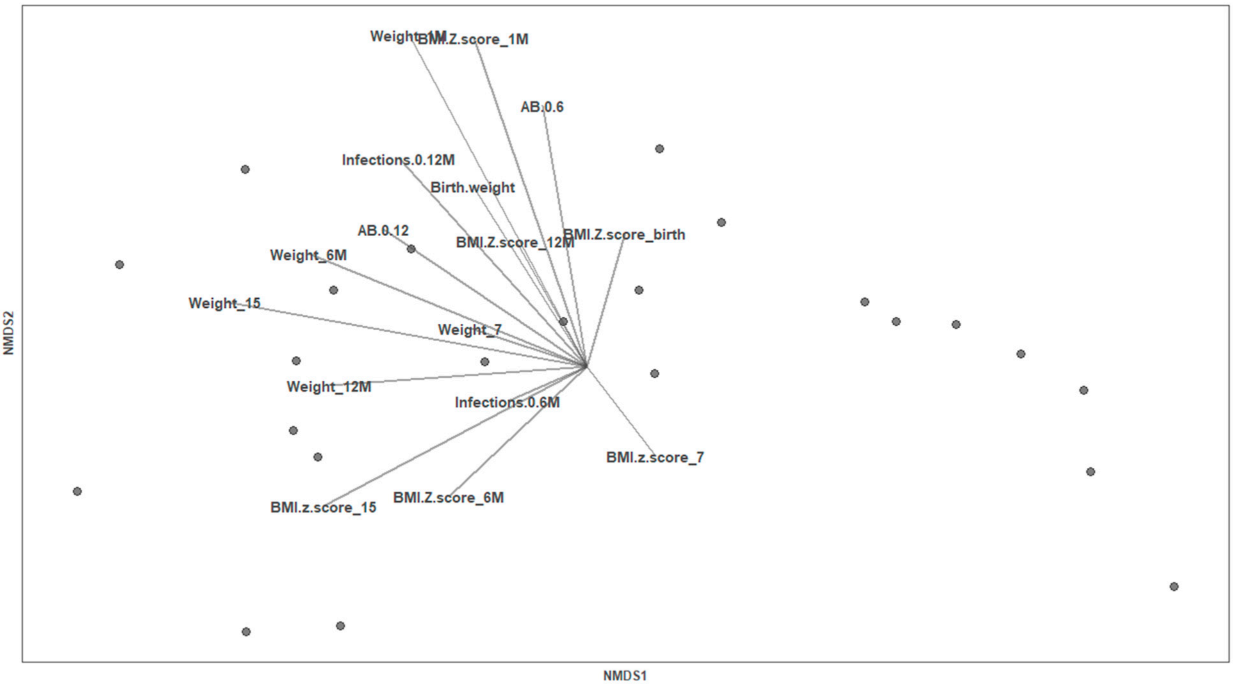

Supplementary Figure S7. Infant factors shaping galectin levels in breastmilk at d15. Non-metric multi-dimensional scaling (NMDS) for the galectin concentrations based on the Bray-Curtis distance (stress: 0.027). Each point represents a mother (n = 23). Continuous variables are represented by vectors.
